# Non-invasive Clinical Measurement of Ocular Rigidity and Comparison to Biomechanical and Morphological Parameters in Glaucomatous and Healthy Subjects

**DOI:** 10.3389/fmed.2021.701997

**Published:** 2021-07-05

**Authors:** Yanhui Ma, Sayoko E. Moroi, Cynthia J. Roberts

**Affiliations:** ^1^Department of Ophthalmology and Visual Sciences, The Ohio State University Wexner Medical Center, Columbus, OH, United States; ^2^Department of Biomedical Engineering, The Ohio State University, Columbus, OH, United States

**Keywords:** ocular rigidity, glaucoma, ocular biomechanics, optical coherence tomography, stiffness parameter, pressure volume relationships

## Abstract

**Purpose:** To assess ocular rigidity using dynamic optical coherence tomography (OCT) videos in glaucomatous and healthy subjects, and to evaluate how ocular rigidity correlates with biomechanical and morphological characteristics of the human eye.

**Methods:** Ocular rigidity was calculated using Friedenwald's empirical equation which estimates the change in intraocular pressure (IOP) produced by volumetric changes of the eye due to choroidal pulsations with each heartbeat. High-speed OCT video was utilized to non-invasively measure changes in choroidal volume through time-series analysis. A control-case study design was based on 23 healthy controls and 6 glaucoma cases. Multiple diagnostic modalities were performed during the same visit including Spectralis OCT for nerve head video, Pascal Dynamic Contour Tonometry for IOP and ocular pulse amplitude (OPA) measurement, Corvis ST for measuring dynamic biomechanical response, and Pentacam for morphological characterization.

**Results:** Combining glaucoma and healthy cohorts (*n* = 29), there were negative correlations between ocular rigidity and axial length (Pearson *R* = −0.53, *p* = 0.003), and between ocular rigidity and anterior chamber volume (*R* = −0.64, *p* = 0.0002). There was a stronger positive correlation of ocular rigidity and scleral stiffness (i.e., stiffness parameter at the highest concavity [SP-HC]) (*R* = 0.62, *p* = 0.0005) compared to ocular rigidity and corneal stiffness (i.e., stiffness parameter at the first applanation [SP-A1]) (*R* = 0.41, *p* = 0.033). In addition, there was a positive correlation between ocular rigidity and the static pressure-volume ratio (P/V ratio) (*R* = 0.72, *p* < 0.0001).

**Conclusions:** Ocular rigidity was non-invasively assessed using OCT video and OPA in a clinic setting. The significant correlation of ocular rigidity with biomechanical parameters, SP-HC and P/V ratio, demonstrated the validity of the ocular rigidity measurement. Ocular rigidity is driven to a greater extent by scleral stiffness than corneal stiffness. These *in vivo* methods offer an important approach to investigate the role of ocular biomechanics in glaucoma.

## Introduction

Accumulating clinical and scientific evidence has confirmed the critical roles of biomechanics in ocular health and disease, specifically in glaucoma (1–4). Glaucoma is the second leading cause of blindness worldwide (5), and represents a significant health and financial burden on the economy. Glaucomatous axonal damage initiates at the optic nerve head (ONH) where the retinal nerve fibers (axons of ganglion cell) exit the eye (6, 7). Mathematical modeling and animal studies have suggested that scleral stiffness is a major determinant of the ONH susceptibility to the damage (8–11). Methods for quantifying the pressure-strain response of the sclera focused mainly on *ex vivo* strip testing and inflation testing (12–14). However, *in vivo* evaluation of scleral stiffness remains limited. Assessing the ocular biomechanics in glaucoma, especially in a clinic setting, is imperative to gain a deeper understanding of tissue behavior using newer technologies.

Ocular rigidity describes the change in intraocular pressure (IOP) in response to a change in ocular volume. The ocular volume fluctuates due to the pulsatile vascular filling that occurs with each heartbeat, and for a given volume change, stiffer eyes will have a correspondingly larger increase in IOP, and vice versa for less stiff eyes (15). The pulsatile IOP change, referred as ocular pulse amplitude (OPA), can be easily measured transcorneally with a pneumotonometer or dynamic contour tonometer (DCT). In contrast, assessing the pulsatile volume change is the challenging part in the process of estimating ocular rigidity. Direct invasive methods involve injecting a known volume of saline solution into the anterior chamber, while continually monitoring the IOP (16), which were only applied to subjects undergoing cataract surgery. Retrobulbar anesthesia during the surgery may alter the ocular rigidity (17). Indirect non-invasive methods involve using the anterior-to-posterior expansion of the corneoscleral shell to estimate the volume change (18, 19), which, however, may obfuscate the measurement of ocular rigidity (20), due to confounding variables, such as the preexisting volume of the choroidal circulation and the preexisting IOP level. Thus, a direct measure of the choroidal volume change produced by blood vessel flux with each cardiac cycle is crucial for the reliable non-invasive estimation of ocular rigidity.

Optical coherence tomography (OCT), as a non-invasive imaging tool for visualizing the cross-section of the retina and choroid with micrometer resolution, has become the standard of care in the ophthalmic field. Recent advances in OCT with enhanced depth imaging have brought previously unavailable insights into choroidal anatomy and pathologies. Choroidal-scleral interface (CSI) can be distinguished from high-quality OCT images permitting the thickness of the choroid to be evaluated. At each heartbeat, the pulsatile vascular filling induces a transient change of the choroidal volume. High-speed OCT with dense temporal sampling (up to 153 frames *per second*) enables us to capture the dynamic response and detect the change in the retina and choroid. We have implemented an automated open-source algorithm for CSI segmentation in sequential OCT images with 599 B-scan frames, which allows for the assessment of pulsatile choroidal thickness change deriving the ocular volume change, then the ocular rigidity in conjunction with an independent measurement of OPA.

This study first aimed to evaluate the ocular rigidity in treated glaucoma patients compared to healthy subjects with our *in vivo* non-invasive approach. Note that this approach for *in vivo* estimation of ocular rigidity would not be easily accessible to clinicians for a foreseeable future due to the fact that not all OCT devices provide time series, and also the custom algorithm is currently limited in the generalizability to the real-world setting. Investigation of how ocular rigidity correlates with clinically-measurable parameters may facilitate identifying the surrogates for *in vivo* ocular rigidity. The second aim of this study is to examine the relationship of ocular rigidity with biomechanical and morphological characteristics of the eye.

## Methods

### Subject Participants and Ophthalmological Examination

All participants have consented in adherence to the tenets of the Declaration of Helsinki. This study was approved by the Institutional Review Board of The Ohio State University. Subjects with a diagnosis of primary open-angle glaucoma by a glaucoma specialist without a history of intraocular surgery were included in the glaucoma cohort. Healthy controls had an untreated IOP lower than 21 mm Hg, healthy discs, and no ocular pathologies. Exclusion criteria for participants included any history of ocular injury and ocular diseases, such as age-related macular degeneration, diabetic retinopathy, keratoconus, retinal detachment, retinal tear, retinal degeneration, or retinal hole. Participants with spherical equivalent refraction < -6 diopters or more than +6 diopters were also excluded. Any OCT images with significant artifactual components due to blockage of OCT signal by floaters and eyelashes, residual motion artifacts, or other artifacts, were excluded from the study to avoid confounding of quantitative analysis.

Data were acquired on multiple ophthalmic diagnostic devices. All patients underwent a complete ophthalmic examination including the Corvis ST (OCULUS, Wetzlar, Germany), Pentacam (OCULUS, Wetzlar, Germany), Pascal DCT (Ziemer, Port, Switzerland), and Spectralis OCT (Heidelberg Engineering, Heidelberg, Germany) examinations during the same visit. Only one eye (right eye) per subject was included in the analysis. Statistical analyses were conducted using the SAS software (V9.4; SAS Institute Inc., Cary, NC, USA). The normality was checked using the Shapiro-Wilk test. The ocular rigidity in the healthy cohort (*n* = 23) was normally distributed, whereas the distribution of ocular rigidity in the glaucoma cohort (*n* = 6) was not normal, likely due to the small sample size. Thus, non-parametric Mann-Whitney U test (also called Wilcoxon rank sum test) was used to compare the data between glaucoma cases and healthy controls. The correlations of ocular rigidity with biomechanical and morphological characteristics of the eye were evaluated using Pearson correlation with groups of healthy and glaucoma subjects combined (*n* = 29).

### Estimation of Ocular Rigidity using Optical Coherence Tomography

**Figure 1:**
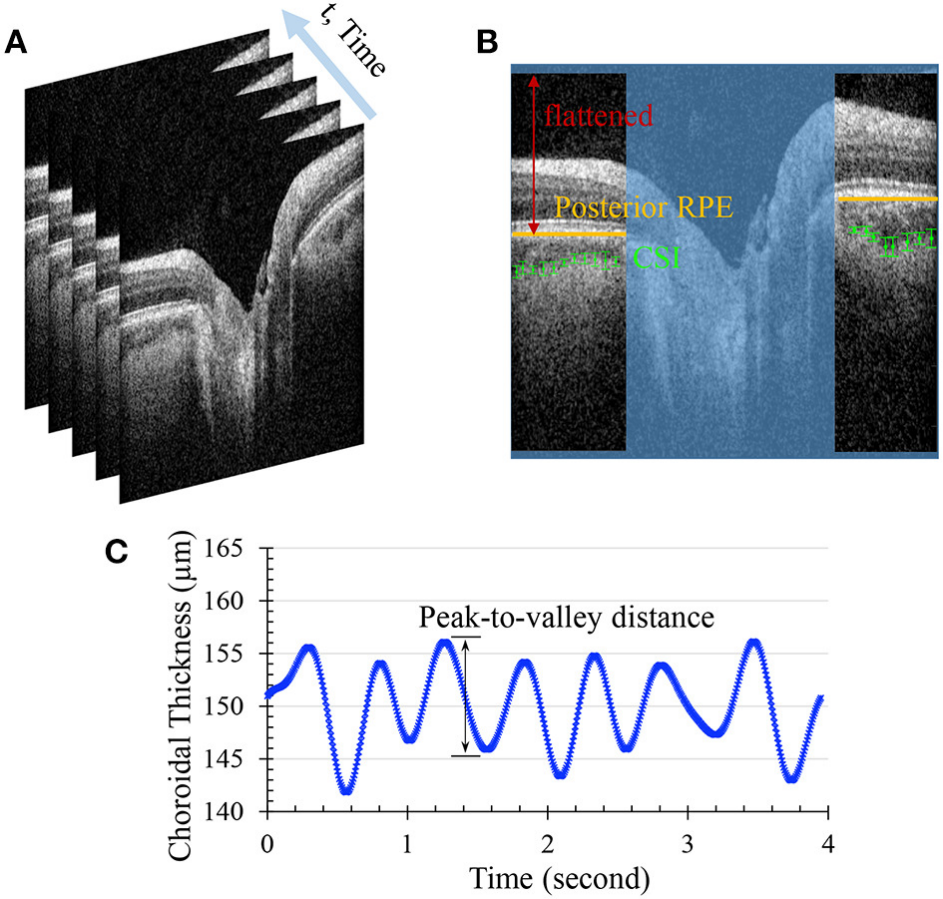
**(A)** Sequential OCT B-scans of the posterior eye centered at the optic nerve head **(B)** Automated segmentation for the choroidal scleral interface (CSI). The optic nerve region was excluded from the region of interest, and the posterior retinal pigment epithelium (RPE) of each side of the optic nerve was flattened before the graph search for CSI nodes. **(C)** Filtered choroidal thickness waveform. The average peak-to-valley distance was calculated as choroidal thickness change.

Spectralis OCT integrated the active eye-tracking (TruTrack) technology to correct eye motion by reacquisition of OCT images at the same retinal location in a fraction of a second. OCT videos of the posterior eye centered at the ONH consisting of 599 B-scan frames were acquired (Figure 1A). Segmentation for the CSI is currently not available on the clinical OCT device that provides time series. Hand-tracing is not only operator-dependent, but also time-consuming and labor-intensive. Herein we have implemented an open-source algorithm for automatically segmenting and quantifying the choroidal layer based on graph search (21). Briefly, graph nodes are defined as the inflection points (where the second derivative changes signs) of the intensity along each depth profile (A-scan). The intensity transition from dark to bright marks the location passing from the choroidal vessel to the sclera. Edge probability was used to compute the weight component for each node (22). Chorioretinal thinning and disruption of the retinal pigment epithelium with the development of peripapillary atrophy (alpha and beta zone of atrophy around the ONH) are more frequently observed in glaucoma (23). It is worth noting that before graph search, each B-scan frame (excluding the central optic nerve region, Figure 1B) is flattened with respect to the posterior retinal pigment epithelium to eliminate erroneous paths resultant of the curvature or tilt of the B-scan. Choroidal thickness (ChT) was then calculated as the average location of all searched nodes subtracted by the posterior retinal pigment epithelium depth at each frame.

Compiling the ChTs of all frames presents ChT fluctuation over time. The period of time for acquisition of 599 B-scans varies in the range of 4–7 s depending on the eye's stability as the built-in eye-tracking feature introduces pauses into the acquisition when the scanning beam could not be held in place due to eye movement. Because of this, the ChT points in the time series are not usually equally spaced. A series of signal process techniques are applied to extract the ChT change. First, ChT values that are more than three median absolute deviations are regarded as outliers and discarded from the waveform. Then the non-equally-spaced data are resampled by incorporating an anti-aliasing filter and compensating for the delay introduced by the filter. In order to extract the ChT fluctuations associated mainly with the heart rate, a band-pass filter is applied to only pass frequencies within the range of 0.5 to 3 times the heart rate. The inverse Fourier Transform is used to retrieve the filtered signal, from which average peak-to-valley distance is calculated as the ChT change (denoted as Δ*t*) (Figure 1C). Since 85% of total ocular blood flow passes through the choroid (24), fluctuation of the ocular volume is estimated by the choroidal volume change. We simplified the choroid as a thin spherical shell, and its volume change is the difference between the volumes of two spheres: $\frac{4}{3}\pi {\left(R+\Delta t\right)}^{3}-\frac{4}{3}\pi {\left(R\right)}^{3}$, where R is approximated by half of the axial length based on a spherical eye model. For a small Δ*t*, the ocular volume change is specified as Δ*V* = 4π*R*^2^Δ*t*. Note that the automated segmentation of high-speed OCT imaging was developed by Beaton et al. (22), and we have independently implemented this approach and extended the image analysis to the ONH region with improvement in signal processing for ChT extraction.

In this study, IOP variation within each cardiac cycle was measured by a Pascal DCT immediately before OCT imaging. It has been reported that DCT is relatively independent of corneal biomechanics, generating accurate and reproducible continuous recording of IOP (25, 26). Finally, the ocular rigidity is estimated using Friedenwald's empirical function, as specified by *lnIOP*_1_−*lnIOP*_2_ = *kΔV*, where *k* denotes the ocular rigidity (15). *IOP*_1_ is the systolic IOP calculated by the sum of IOP reading and OPA from DCT measurement. *IOP*_2_ is the diastolic IOP provided directly by the IOP reading from DCT.

### Corneoscleral Biomechanical Response Induced by Air Puff

Dynamic corneal response parameters were measured by Corvis ST which is a novel, non-contact, tonometer coupled with a high-speed Scheimpflug camera that allows investigation of the dynamic reaction of the cornea to an air impulse. The camera acquires 140 sequential images of the central cornea with 8 mm horizontal coverage at over 4,330 frames *per second*. Good repeatability and reproducibility have been shown for dynamic corneal response parameters (27). Stiffness parameters at the first applanation (SP-A1) and highest concavity (SP-HC) were derived from the directly measured dynamic corneal response parameters (28). Specifically, SP-A1 was calculated by (*AP*1_*adj*_−*bIOP*)/δ_*A*1_, where *AP*1_*adj*_ is the adjusted air pressure at the time of first applanation, *bIOP* is biomechanically corrected intraocular pressure (29), and δ_*A*1_ is the deflection at first applanation. SP-HC was calculated by (*AP*1_*adj*_−*bIOP*)/(δ_*HC*_−δ_*A*1_), where δ_*HC*_ is the maximum deflection near the highest concavity. In this study, the corneoscleral biomechanical properties characterized from Corvis ST, namely SP-A1 and SP-HC for corneal stiffness and scleral stiffness, respectively, were examined and correlated with ocular rigidity estimated using OCT.

### Ocular Characteristics

The Pentacam was used to measure the radius of corneal curvature, central cornea thickness (CCT), and anterior chamber volume (ACV). IOP and OPA were measured with the Pascal DCT. A direct measure of axial length (AL) was not available for all subjects in this study. We therefore derived AL from the Gullstrand-Emsley model (30, 31) using the focus setting (refraction) on Spectralis and the radius of corneal curvature. To validate this approach, a separate group of subjects (*n* = 53 eyes from healthy and pathological subjects) was used that had measurements from both the Spectralis and the ANTERION (Heidelberg Engineering, Heidelberg, Germany). We compared the calculated AL using the Spectralis with that directly reported by the ANTERION using Bland-Altman analysis.

## Results

### Ocular Characteristics in Healthy Controls vs. Glaucoma Cases

**Figure 2:**
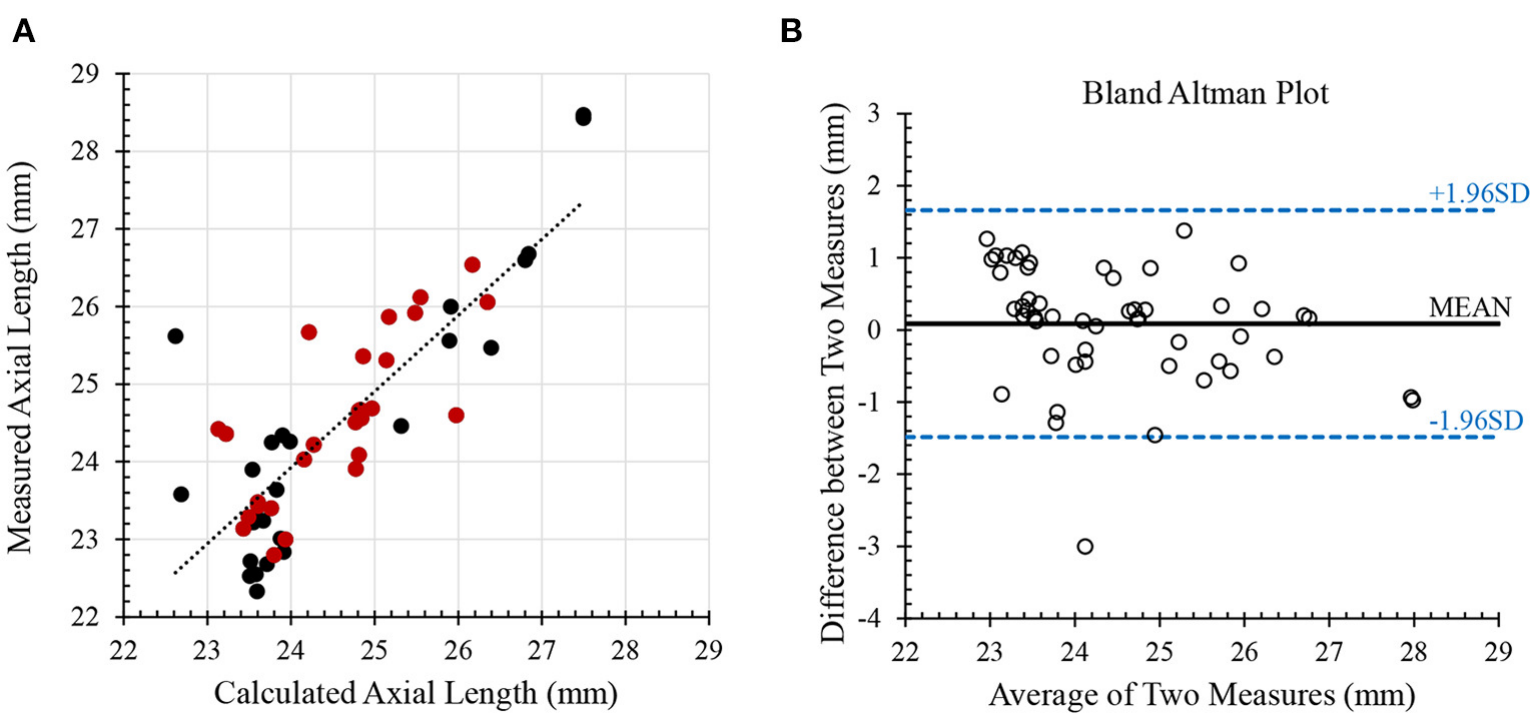
Validation of axial length. **(A)** the calculated axial length from Spectralis was strongly correlated with the measured axial length from ANTERION. Red dots indicate healthy subjects and black dots indicate pathological subjects (*n* = 53; Pearson *R* = 0.83; *p* < 0.00001) **(B)** Bland-Altman plot for comparing calculated and measure axial lengths.

Twenty-nine subjects with processable OCT videos and valid IOP measurements were ultimately included in this study (23 healthy controls and 6 glaucoma cases). Demographics and ocular characteristics for patients with glaucoma cases and healthy eye controls are summarized in Table 1. The calculated AL was strongly correlated with the measured AL (*p* < 0.00001, Figure 2A), and the paired *t*-test suggested that there was no statistically significant difference (*p* > 0.1), validating our approach for axial length assessment using the OCT device. Bland-Altman plot for calculated AL and measured AL is shown in Figure 2B.

**Table 1:** Demographic and ocular characteristics of all subjects (*n* = 29).

	**Control (*n* = 23)**	**Glaucoma (*n* = 6)**	***p*-value**
Age (*years*)	40.0 ± 12.6	61.5 ± 8.4	0.002*
Central corneal thickness (*μm*)	557.90 ± 32.18	544.22 ± 22.93	0.32
Radius of corneal curvature (*mm*)	7.76 ± 0.22	7.65 ± 0.38	0.39
Axial length (*mm*)	24.82 ± 0.89	24.62 ± 1.22	0.65
Anterior Chamber volume (*μL*)	175.30 ± 30.08	167.72 ± 41.55	0.98
Intraocular pressure (*mmHg*)	16.95 ± 2.00	20.79 ± 6.41	0.07
Ocular pulse amplitude (*mmHg*)	2.46 ± 1.14	3.49 ± 1.51	0.12
Ocular volume change (*μL*)	9.79 ± 3.29	8.16 ± 1.73	0.35
Ocular rigidity (*μL^−1^*)	0.015 ± 0.005	0.020 ± 0.010	0.29
SP-A1^‡^	129.06 ± 14.90	128.04 ± 17.92	0.93
SP-HC^‡^	15.23 ± 3.41	15.23 ± 5.10	0.70

Differences between healthy and glaucoma subjects were evaluated by using Mann–Whitney U-tests with a significant level of 0.05. Asterisk (*) indicates a significant difference.

‡ *Sample size for SP-A1 and SP-HC is 21 for control and 6 for glaucoma*.

No significant difference was found in CCT, ACV, AL, radius of corneal curvature, ocular volume change, DCT-measured IOP, and OPA between treated glaucoma subjects and healthy controls using non-parametric Mann–Whitney *U*-test (Table 1). The glaucoma cohort was older than subjects in the healthy control cohort (61.5 ± 8.4 years vs. 40.0 ± 12.6 years; *p* = 0.002). Ocular rigidity was not correlated with age in this dataset with *n* = 29 combing healthy and glaucoma cohorts (Pearson *R* = 0.06; *p* = 0.75).

The mean ocular rigidity in the 23 healthy controls was 0.015 μL^−1^ (95% confidence interval, 0.012 to 0.017 μL^−1^), and the mean ocular rigidity in the 6 glaucoma cases was 0.020 μL^−1^ (95% confidence interval, 0.009 to 0.030 μL^−1^). Ocular rigidity did not demonstrate a significant difference between the treated glaucoma subjects and healthy controls (*p* = 0.29). The dynamic corneal response parameters measured by Corvis ST were not available in two subjects (out of 23) in the control cohort. The corneoscleral stiffness parameters, namely SP-A1 and SP-HC, were compared between 6 glaucoma and 21 healthy subjects. No significant difference was observed in glaucoma compared to healthy cohorts in terms of the corneoscleral stiffness parameters in this dataset. Table 1 summarizes the mean value and standard deviation of all the ocular characteristics in the treated glaucoma cohort and healthy cohort, and their comparison *p*-value using the Mann–Whitney *U*-test.

### Ocular Rigidity vs. Morphological Characteristics and Stiffness Parameters

**Figure 3:**
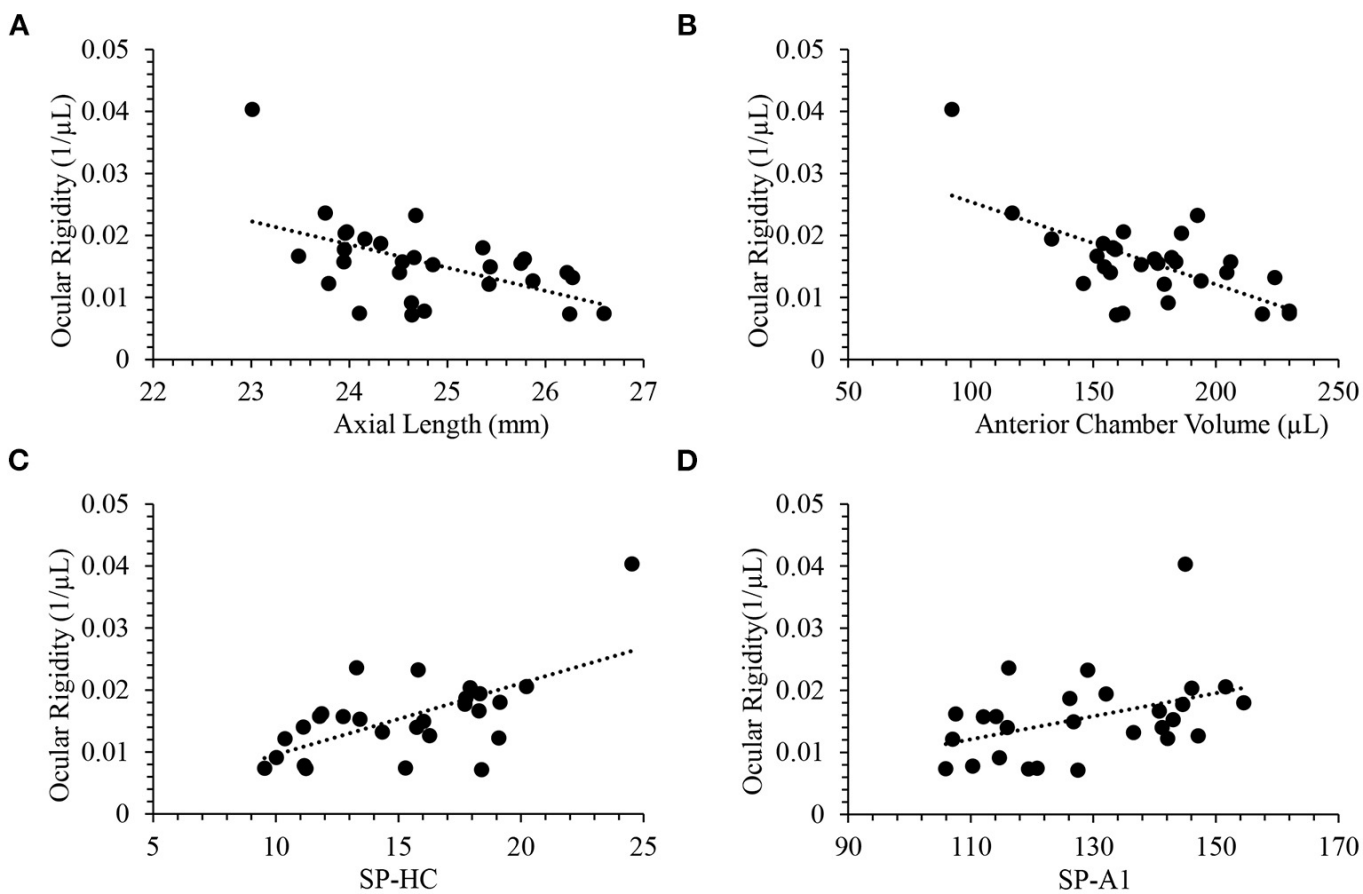
Ocular rigidity was negatively correlated with **(A)** axial length (*R* = −0.53; *p* = 0.003) and **(B)** anterior chamber volume (*R* = −0.64; *p* = 0.0002). Ocular rigidity was positively correlated with **(C)** SP-HC (*R* = 0.62; *p* = 0.0005) and **(D)** SP-A1 (*R* = 0.41; *p* = 0.033).

With glaucoma and healthy cohorts combined (*n* = 29), there were negative correlations between ocular rigidity and AL (*R* = −0.53, *p* = 0.003, Figure 3A), and between ocular rigidity and ACV (*R* = −0.64, *p* = 0.0002, Figure 3B); while ocular rigidity was not correlated with CCT or radius of corneal curvature. There was a positive correlation between ocular rigidity and OPA (*R* = 0.51, *p* = 0.004); whereas there was no correlation between ocular rigidity and IOP. For the biomechanical parameters, ocular rigidity was shown to be positively correlated with SP-HC (*R* = 0.62, *p* = 0.0005, Figure 3C) and SP-A1 (*R* = 0.41, *p* = 0.033, Figure 3D). This correlation analysis was determined with the combination of the healthy cohort (*n* = 23) and glaucoma cohort (*n* = 6). When only the healthy cohort was included, the results of correlation analysis were consistent. The parameters that demonstrated a significant correlation with ocular rigidity in the healthy cohorts included AL (negative), ACV (negative), OPA (positive), SP-A1 (positive), and SP-HC (positive). The Pearson correlation coefficients between ocular rigidity and ocular characteristics are tabulated in Table 2.

**Table 2:** Correlation of ocular rigidity with morphological characteristics and stiffness parameters.

	**Glaucoma** **+** **control (*****n*** **=** **29)**		**Control only (*****n*** **=** **23)**	
**Correlation of ocular rigidity with:**	**R**	***p*-value**	**R**	***p*-value**
Central corneal thickness	0.069	0.72	0.083	0.71
Radius of corneal curvature	−0.089	0.65	0.20	0.36
Axial length	−0.53	0.003*	−0.44	0.034*
Anterior chamber volume	−0.64	0.0002*	−0.50	0.015*
Intraocular pressure	0.079	0.68	−0.070	0.75
Ocular pulse amplitude	0.51	0.004*	0.41	0.049*
SP-A1^‡^	0.41	0.033*	0.43	0.050*
SP-HC^‡^	0.62	0.0005*	0.48	0.026*

Asterisk (*) indicates a significant correlation at the level of 0.05.

‡ *Sample size for SP-A1 and SP-HC is 21 for control and 27 for glaucoma and control combined*.

## Discussion

The ocular rigidity estimates the change in IOP produced by volumetric changes of the eye due to choroidal pulsations. We have implemented an approach for direct non-invasive measurement of choroidal volume change through automated segmentation using high-speed OCT that incorporates time series. To assess the role of ocular rigidity in glaucoma, the OCT videos in this study were taken at the ONH. This non-invasive approach for estimation of choroidal volume change was reported by another group recently that validated using sequential OCT imaging centered at the macula, and the repeatability was found to be good with an intra-session correlation coefficient of 0.96 (22, 32). We have found a statistically significant negative correlation between ocular rigidity and axial length (Figure 3A). A previous study that estimated the ocular rigidity invasively by monitoring the IOP change caused by the injection of saline solution also showed the negative correlation between ocular rigidity and axial length (33). Our findings on ocular rigidity determined using a non-invasive approach were in the same range as those reported in early studies using an invasive approach (16, 33). Thus, the consistency between the ocular rigidity measurements determined by an invasive approach and our ocular rigidity data determined by our non-invasive approach provides evidence of the validity of the measurements.

Quantitative characteristics of corneal biomechanical parameters derived from Corvis ST have demonstrated diagnostic power in corneal disease (34). The stiffness parameter at the first applanation SP-A1 is indicative of corneal stiffness (28). A finite-element study on the biomechanical impact of the sclera on displacement amplitude reported that a stiffer sclera limits corneal deformation (35). It was suggested that the stiffness parameter at the highest concavity (SP-HC) is indicative of scleral stiffness, as validated by *ex vivo* experiments, in which SP-HC was found to be significantly higher after scleral stiffening with 4% glutaraldehyde without changes in corneal parameters (36). Thus, SP-HC derived from air-puff induced deformation offers a clinical measure indicating scleral stiffness. Although our results showed no significant difference in SP-HC between healthy and glaucoma subjects due to small *n*, a positive correlation of SP-HC was found with ocular rigidity (Figure 3C) despite the small sample size. Note that the ocular rigidity accounts for the properties of both the sclera and cornea. Since the cornea is less stiff than the sclera and the sclera encompasses greater surface area than the cornea, the ocular rigidity is driven to a greater extent by scleral stiffness than corneal stiffness in normal and disease. This statement is supported by the evidence that our data showed a stronger correlation of ocular rigidity with SP-HC than with SP-A1 (Table 2, Pearson *R* = 0.62 vs. 0.41). These parameters are analogous to others in combined analyses of controls and glaucoma cases that have been successfully investigated as a continuous quantitative trait in genome-wide association studies that investigated the risk factor for glaucoma, such as CCT (37) and IOP (38).

A previous study has reported a positive correlation between ocular rigidity and age in 79 living human eyes, in which ocular rigidity was determined by cannulating the anterior chamber in patients undergoing cataract surgery (16). In the current study, no correlation between ocular rigidity and age was observed, possibly due to the smaller sample size (*n* = 29) and the different approaches to quantifying ocular rigidity. Inflation tests of human eyes have shown the age-related stiffening in the pressure-strain response in the sclera (39, 40). This observed tissue behavior may be due to a mechanism related to an accumulation of intermolecular non-enzymatic cross-linking (4). As it has been speculated that the scleral stiffness increases with age, the fact that open-angle glaucoma prevalence increase with age may be sharing a mechanism of scleral stiffness as part of the pathogenesis of glaucoma. Computational and *ex vivo* experimental studies have demonstrated that the sclera becomes stiffer with glaucoma (41, 42). Whether or not a stiffer sclera is a risk factor for glaucoma remains unclear and inconclusive. Based on computational models representing generic ONH geometry and material properties (8), higher sclera stiffness was associated with less deformation in ONH tissues. On the other hand, mechanical insult has been hypothesized as an initiating factor and a driving force in the disease process of glaucoma (43, 44), suggesting more ONH deformation may be related to more glaucomatous axonal damage. Lower ocular rigidity was found to be positively correlated to greater glaucomatous damage represented by ganglion cell complex, retinal nerve fiber layer thicknesses, and neuroretinal rim area (45). It must be acknowledged that there are multiple contributing factors to the pathogenesis of glaucoma, and the change of biomechanical environment could drive connective tissue remodeling, possibly resulting in an alteration of stiffness in the progression of glaucoma (2). In addition, the use of prostaglandin analogs (PGA) for the treatment of glaucoma may lead to changes in the biomechanical properties of the eye (46).

**Figure 4:**
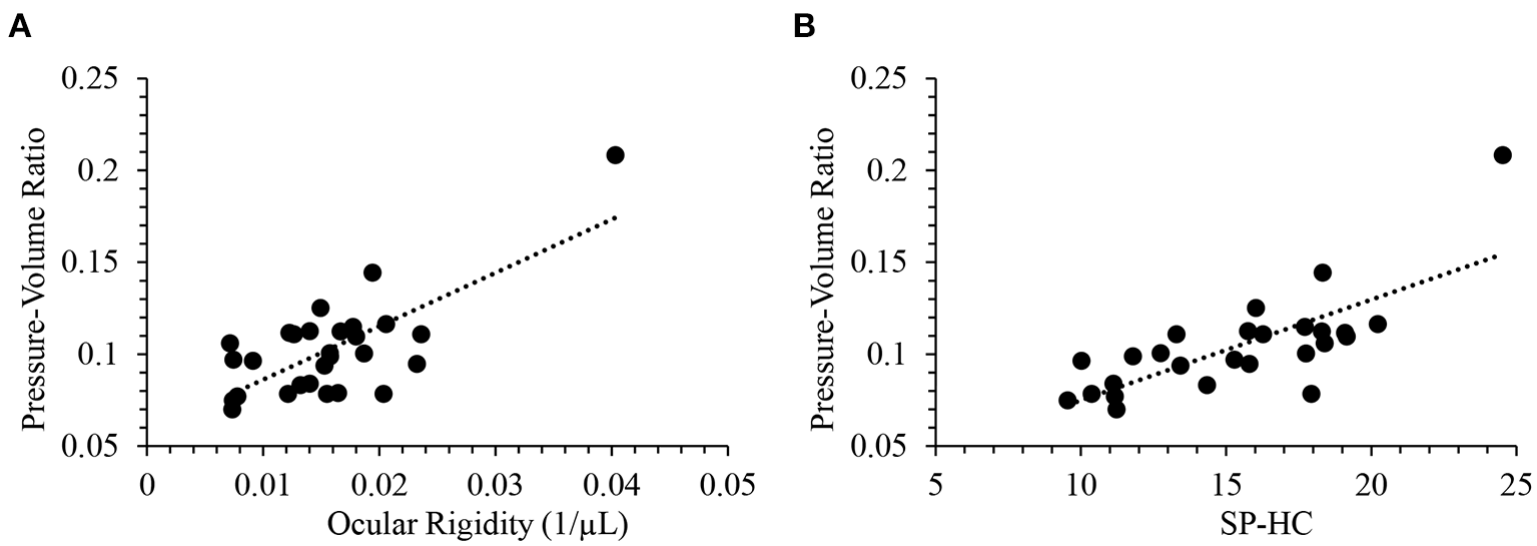
Pressure-volume ratio was positively correlated with **(A)** ocular rigidity (*R* = 0.72; *p* < 0.0001) and **(B)** SP-HC (*R* = 0.75; *p* < 0.0001).

IOP fluctuation over time may be a result of both physiological regulations related to a circadian rhythm of aqueous humor secretion, and progressive damage to the segmental outflow through the trabecular meshwork. The ocular rigidity based on Friedenwald's empirical equation reveals the pressure-volume relationship in the eye that takes the fluctuation into consideration. Ocular compliance defined as Δ*V*/Δ*P* was previously measured in mice using iPerfusion which also accounts for the dynamic mechanical response of the eye (47). Alternatively, a simple static pressure-volume relationship could be the pressure-volume ratio (P/V ratio) calculated as IOP divided by ACV. There was a strong positive correlation between ocular rigidity and P/V ratio (Pearson R= 0.72; *p* < 0.0001). Figure 4A provides a scatterplot of the relationship between ocular rigidity and P/V ratio. In addition, P/V ratio and SP-HC were significantly correlated (*R* = 0.75; *p* < 0.0001, Figure 4B). Ocular rigidity estimated from OCT (plus Pascal DCT for IOP measurement), SP-HC quantified from Corvis ST, and P/V ratio characterized by Pentacam (plus DCT) correlated with each other. To the best of our knowledge, our study design is one of the first reports to evaluate the association of different ocular biomechanical parameters measured from multiple ophthalmic devices.

IOP remains the only modifiable and treatable risk factor for the development and progression of glaucoma. Studies have confirmed the benefit of lowering IOP in glaucoma patients, even in those without detectable high IOP (48, 49). PGAs have been used as first-line monotherapies for IOP reduction in adult patients with glaucoma. A recent prospective study evaluated the relationship of IOP and ACV with the use of PGAs in glaucoma patients, and it suggested that P/V ratio before the naïve use of PGA therapy (baseline visit) was significantly correlated with the IOP reduction at visit 2 (1 month after the naïve use of PGA) (46). It reported that the majority of eyes had a decrease in ACV with a decrease in IOP after the use of PGAs. Paradoxically, it also showed that in one-third of treated glaucoma eyes, a mean increase in ACV was accompanied by a mean decrease in IOP (46), suggesting that ocular rigidity is altered after treatment with PGA therapy. The chronic use of PGAs has shown to be associated with a decrease in the collagen type I level (50, 51), which is the main load-bearing constituent of the extracellular matrix existing in human eye tissues, such as cornea and sclera. The mechanism of changing ocular rigidity after the naïve use of PGA therapy warrants further investigation.

Limitations of this study include that the method for choroidal volume change estimation is highly dependent on the image quality of OCT video. Different from the structural OCT image which relies on real-time image averaging of multiple B-scans to enhance signal-noise ratio, no averaging was set for the acquisition of OCT video in the current study. In addition, since the central optic nerve region with no choroid was excluded from the analysis, a relatively small region of the image was available for processing. The simplification of volume change based on the choroidal thickness change, Δ*t*, at a single cross-sectional scan limits its ability to incorporate the possible difference in Δ*t* at different regions, such as macula vs. ONH, nasal-temporal vs. superior-inferior. Peripapillary choroidal volumetric parameters may be impacted by the alpha- and beta-zone around the ONH in glaucoma (23), however, it is unclear if the pulsatile volume change is altered by the peripapillary atrophy. The mathematical model used to extrapolate the pulsatile ocular volume change was based on a spherical eye model, which simplified the process for ocular rigidity estimation, but was limited in accounting for anatomical characteristics of the choroid. Another limitation was the small sample size in the glaucoma cohort. New algorithms for improving the CSI segmentation and choroidal volume change estimation are currently being developed with the objective of processing more OCT videos and increasing the sample size.

In conclusion, non-invasive clinical measurement of ocular rigidity was determined using sequential OCT imaging and OPA measurement. No significant difference in ocular rigidity was detected in the treated glaucoma subjects and healthy controls. As the measured ocular rigidity describes the total response of the eye, it was found to be correlated with ocular morphological and biomechanical characteristics. Specifically, there were negative correlations between ocular rigidity and axial length, and between ocular rigidity and anterior chamber volume. In addition, ocular rigidity significantly increased with increasing corneoscleral stiffness parameters characterized by air-puff induced deformation. The significant correlation of ocular rigidity with SP-HC and pressure-volume ratio demonstrated not only the validity of this measurement, but also the consistency of multiple ophthalmic devices in examining ocular biomechanics. A larger longitudinal study may provide greater insights into the development and progression of glaucoma, and response to treatment.

## Data Availability Statement

The datasets presented in this article are not readily available because the datasets will not be available until after the grant concludes. Requests to access the datasets should be directed to Cynthia Roberts, roberts.8@osu.edu.

## Ethics Statement

The studies involving human participants were reviewed and approved by the Institutional Review Board of The Ohio State University. The patients/participants provided their written informed consent to participate in this study.

## Author Contributions

YM: algorithm implementation and improvement, analysis and interpretation of data, writing, and revising the manuscript. SM: interpretation of data and critical revision of the manuscript. CR: conception and design, interpretation of data, and critical revision of the manuscript. All authors approved the submitted version.

## Conflict of Interest

SM holds a patent that is not related to this work and receives royalties from Wolters Kluwer Health that is not related to this work. CR is a consultant to OCULUS Optikgerate GmbH and Zeimer Ophthalmic Systems AG. The remaining author declares that the research was conducted in the absence of any commercial or financial relationships that could be construed as a potential conflict of interest.
